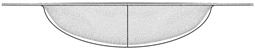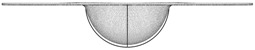# Thermomechanical Modeling of Amorphous Glassy Polymer Undergoing Large Viscoplastic Deformation: 3-Points Bending and Gas-Blow Forming

**DOI:** 10.3390/polym11040654

**Published:** 2019-04-10

**Authors:** Jun Wang, Yingjie Xu, Weihong Zhang, Xuanchang Ren

**Affiliations:** 1 Intelligent Materials and Structures, Unmanned System Research Institute, Northwestern Polytechnical University, Xi’an 710072, China; wang.jun@nwpu.edu.cn; 2 State IJR Center of Aerospace Design and Additive Manufacturing, Northwestern Polytechnical University, Xi’an 710072, China; renxuanchang@163.com; 3 Shaanxi Engineering Laboratory of Aerospace Structure Design and Application, Northwestern Polytechnical University, Xi’an 710072, China

**Keywords:** amorphous glassy polymer, constitutive model, viscoplasticity, three-point bending, gas-blow forming

## Abstract

Polymeric products are mostly manufactured by warm mechanical processes, wherein large viscoplastic deformation and the thermomechanical coupling effect are highly involved. To capture such intricate behavior of the amorphous glassy polymers, this paper develops a finite-strain and thermomechanically-coupled constitutive model, which is based on a tripartite decomposition of the deformation gradient into elastic, viscoplastic, and thermal components. Constitutive equations are formulated with respect to the spatial configuration in terms of the Eulerian Hencky strain rate and the Jaumann rate of Kirchhoff stress. Hyperelasticity, the viscoplastic flow rule, strain softening and hardening, the criterion for viscoplasticity, and temperature evolution are derived within the finite-strain framework. Experimental data obtained in uniaxial tensile tests and three-point bending tests of polycarbonates are used to validate the numerical efficiency and stability of the model. Finally, the proposed model is used to simulate the gas-blow forming process of a polycarbonate sheet. Simulation results demonstrate well the capability of the model to represent large viscoplastic deformation and the thermomechanical coupling effect of amorphous glassy polymers.

## 1. Introduction

Amorphous glassy polymers are a group of thermoplastics that do not crystallize below the glass transition temperature $T_{g}$. Their extraordinary physical (lightweight), optical (light transparency), and mechanical (toughness, impact resistance) properties allow them to be widely used in a variety of engineering applications, such as aircraft canopies, explosion shields, goggles, medical apparatuses, etc. [1,2]. These polymeric products are mostly manufactured by warm mechanical processes such as extrusion, drawing, blow forming, and calendering, wherein glassy polymers undergo large viscoplastic deformations and exhibit the thermomechanical coupling effect [3,4]. Thus, to capture such intricate behavior accurately, developing a reliable and practical constitutive model is of prime significance for the design and manufacture of polymeric products.

Over the last few decades, considerable effort has been devoted to developing constitutive models that can describe viscoelastic and large viscoplastic deformations of amorphous glassy polymers [5,6,7,8,9]. Among these models, the “Boyce–Parks–Argon (BPA)” model, The “Oxford-Glass-Rubber (OGR)” model, and the “Eindhoven-Glassy-Polymer (EGP)” model are representative and frequently referenced. The BPA model was developed by the research group of Mary Boyce at MIT based on the mechanisms of molecular motions [3,10,11]. It assumes that the viscoplastic flow and hardening behavior are related to the chain-segment rotation and the chain alignment. The stress in polymer is physically attributed to the entropic resistance to chain alignment and intermolecular resistance to chain-segment rotation. The OGR model was developed by the research group of Paul Buckley at Oxford [12,13,14]. The basic assumption of this model is that the strain energy comes from two mechanisms: perturbation of interatomic potentials (bond stretching) and perturbation of configurational entropy through the change of molecular conformations. The EGP model was developed by the research group of Leon Govaert in Eindhoven [15,16,17]. The basis of this model is the split of the total stress into two contributions: the strain hardening attributed to the molecular orientation of the entanglement network and the plastic flow attributed to intermolecular interaction. Recently, Anand et al. developed an elasto-viscoplasticity theory for finite deformations of amorphous glassy polymers based on the principle of virtual power and used it to simulate the thermomechanical response of polymeric components and devices [4,18]. Bouvard et al. proposed an internal state variable material model based on a hierarchical multiscale approach to predict the time, temperature, and stress state dependence of amorphous glassy polymers [19,20,21]. Fleischhauer et al. developed a 3D constitutive model to represent the thermomechanical behavior of glassy polymers, the rheology of which consists of a Langevin-type free energy function due to molecular alignment, a Maxwell element, and a viscoplastic dashpot [22]. Nada et al. introduced a new concept of a “molecular chain slip system” and presented a molecular chain plasticity model to describe large inelastic deformation of glassy polymers [23]. Bai et al. put forward a hyper-viscoelastic constitutive model for polyurea by separating the rate-independent hyperelasticity and rate-dependent viscoelasticity according to the strain rate magnitude [24], which is in fact a combination of the Ogden model and the K-BKZ model.

In Wang et al. (2016) [25], an elastic-viscoplastic constitutive model for amorphous glassy polycarbonate was developed to represent the strain rate- and temperature-dependent yielding, damage-induced softening, and viscoplastic hardening. Damage evolution associated with the decreasing elastic modulus was highlighted in experiments. The model was established based on a hypothetical molecular unit and a rheological model. The nonlinear yield transition and strain softening and hardening are physically related to the disentanglement, reconfiguration, and alignment of polymer chains. In Wang et al. (2018) [26], a thermomechanically-coupled constitutive model was developed to describe the dynamic behavior of glassy polymer subjected to strong impact loadings. FE simulation of the high-velocity ballistic impact tests on a polycarbonate sheet were carried out using the proposed model. The preliminary works showed the capability to capture primary characteristics of glassy polymer over a wide range of temperatures and strain rates. Nevertheless, the finite-strain and thermodynamically-consistent framework was not yet established.

To this end, this paper aims to develop a new thermomechanical model that can adequately represent large viscoplastic deformation of amorphous glassy polymer. The model is formulated using the finite Hencky strain measure, which has two advantages: first, the finite Hencky strain can be additively split into pure spherical and deviatoric parts, in analogy to the infinitesimal strain formulation [27]; then, the Eulerian Hencky strain rate is conjugated to the Jaumann rate of Kirchhoff stress, which is of great use when formulating the model with respect to the spatial configuration [28]. Moreover, in order to describe the thermal expansion and thermomechanical coupling effect during the warm mechanical process reasonably, the model introduces thermal deformation in addition to the elastic and viscoplastic deformations.

The paper is organized as follows: Section 2 details the derivation of the constitutive model. Section 3 validates the proposed model against experimental data. Section 4 gives an example of a polymeric beam subjected to three-point bending. Section 5 simulates the gas-blow forming process of a polymer sheet using the proposed model. Finally, Section 6 draws the conclusion of this study.

## 2. Results

In most of the available literature, the viscoplastic response of amorphous polymers is modeled following a well-established finite-strain elastoplasticity approach, wherein the deformation gradient is simply decomposed into elastic and plastic parts, i.e., $F=F^{e}F^{p}$. Here, in order to further account for the thermomechanical coupling effect, we assumed a tripartite decomposition of the deformation into elastic, viscoplastic, and thermal components [29]

(1) $$F=F^{e}F^{p}F^{\theta },$$

where $F^{e}$ is defined with respect to an intermediate stress-free configuration, $F^{p}$ with respect to a thermally-expanded configuration, and $F^{\theta }$ with respect to the reference configuration.

Here, with regard to the viscoplastic and thermal deformations, we made the following kinematic hypotheses:

- the viscoplastic deformation is incompressible and irrotational, namely the determinant of the viscoplastic deformation equals one $J_{p}=\det F^{p}=1$, and the viscoplastic stretching tensor equals the velocity gradient of the viscoplastic deformation $D_{p}={\dot{F}}^{p}{F^{p}}^{-1}$ [30,31];
- the thermal deformation is assumed to be an isotropic thermal expansion, i.e., $F_{\theta }=J_{\theta }^{\frac{1}{3}}1$, where 1 denotes the second-order identity tensor.

By the combination of the above multiplicative decomposition and kinematic hypotheses, we obtained the corresponding additive split of the stretching tensor into elastic, viscoplastic, and thermal parts:

(2) $$D={\bar{D}}^{e}+{\tilde{D}}^{p}+\frac{1}{3}\dot{\delta }1,$$

where ${\bar{D}}^{e}$ denotes the deviatoric elastic stretching tensor, ${\tilde{D}}^{p}=R^{e}D^{p}{R^{e}}^{T}$ denotes the spatially-rotated viscoplastic stretching tensor, and $\dot{\delta }$ denotes the rate of the volumetric deformation due to the thermal expansion.

### 2.1. Hyperelasticity

Using the definition of co-rotational rate [32], we can express the hyperelastic relation in terms of the Eulerian rate formulation:

(3) $$\overset{˚}{\tau }=2\mu {\bar{D}}^{e}+K\left(\dot{\delta }-3\alpha \dot{\theta }\right)1,$$

where $\overset{˚}{\tau }=\dot{\tau }-W\tau +\tau W$ denotes the Jaumann rate of the Kirchhoff stress τ, α is the thermal expansion coefficient, and μ and *K* are the shear and bulk moduli, which depend on Poisson’s ratio ν and Young’s modulus *E*. According to the experimental findings [25], we postulate that Poisson’s ratio ν is a constant below the glassy transition temperature, while Young’s modulus *E* varies linearly with the temperature as:

(4) $$E=\Delta E_{\theta }\left(\theta -\theta_{0}\right)+E_{0},$$

where $E_{0}$ denotes the elastic modulus at the reference temperature $\theta_{0}$ and $\Delta E_{\theta }$ is a model parameter determining the variation of the elastic modulus with the temperature. Between the glass (α) and secondary (β) transition temperatures, $\Delta E_{\theta }$ denotes the slope of the modulus-temperature line [26]. According to the literature [33], the variation of the elastic modulus with the temperature can be physically attributed to the failure of secondary bonds during relaxation. Therefore, in one sense, the parameter $\Delta E_{\theta }$ indicates the failure degree of the secondary bonds.

Applying the co-rotational integration to Equation (3) [32], we obtain:

(5) $$\tau =\int_{c}\overset{˚}{\tau }\mathrm{dt}\;\mathrm{and}\;{\bar{h}}^{e}=\int_{c}{\overset{˚}{\bar{h}}}^{e}\mathrm{dt}=\int_{c}{\bar{D}}^{e}\mathrm{dt},$$

where $\int_{c}$ denotes the co-rotational integration operator and ${\overset{˚}{\bar{h}}}^{e}={\dot{\bar{h}}}^{e}-\Omega {\bar{h}}^{e}+{\bar{h}}^{e}\Omega$ denotes the logarithmic co-rotational rate of the Eulerian Hencky strain.

With the substitution of Equation (5) into Equation (3), we obtained the integral form of the hyperelasticity:

(6) $$\tau =2\mu {\bar{h}}^{e}+K\left[\delta -3\alpha (\theta -\theta_{0})\right]1.$$

### 2.2. Viscoplastic Flow Rule

Following the principle of maximum dissipation [26], we introduce an associative flow rule to guarantee the nonnegative dissipation with respect to the irreversible viscoplastic deformation:

(7) $${\tilde{D}}^{p}=\dot{\gamma }\sqrt{\frac{3}{2}}\frac{s}{∥s∥},$$

where $\dot{\gamma }$ is a nonnegative viscoplastic multiplier and s denotes the deviatoric component of the Kirchhoff stress tensor τ.

### 2.3. Strain Softening

The strain softening behavior of an amorphous polymer refers to the evident post-yield stress drop when the material initially enters the viscoplastic regime. It can be physically attributed to the transient disordering of the polymeric networks and the rearrangement of the material defects. Following the idea of [3,18], we introduce an internal state variable $\zeta_{s}$ to describe the strain softening behavior, the evolution equation of which is given as:

(8) $${\dot{\zeta }}_{s}=\left(\chi -\beta_{s}\zeta_{s}\right)\dot{\gamma }\;\mathrm{with}\;\dot{\chi }=-\beta_{s}\chi \dot{\gamma }\;\mathrm{and}\;\chi \left(0\right)=\chi_{0}$$

where the model parameters $\beta_{s}$ and $\chi_{0}$ depend on the temperature as:

(9) $$\beta_{s}=c_{1}\left(\theta -\theta_{0}\right)+c_{2}\;\mathrm{and}\;\chi_{0}=c_{3}\left(\theta -\theta_{0}\right)+c_{4}$$

where $c_{1}$, $c_{2}$, $c_{3}$, and $c_{4}$ are material constants.

### 2.4. Strain Hardening

With the increasing strain, the strain softening fades away, while the subsequent strain hardening begins to dominate the viscoplastic behavior. This phenomenon is physically attributed to the alignment of polymeric chains. Likewise, we introduce an internal state variable $\zeta_{h}$ to describe the strain hardening behavior, the evolution equation of which is given as:

(10) $${\dot{\zeta }}_{h}=\beta_{h}{\bar{h}}_{p}\left(1-\frac{\zeta_{h}}{\chi_{\infty }}\right)\dot{\gamma }$$

where ${\bar{h}}_{p}=\int \dot{\gamma }\mathrm{dt}$ denotes the cumulative viscoplastic strain, and the model parameters $\beta_{h}$ and $\chi_{\infty }$ depend on the temperature as:

(11) $$\beta_{h}=c_{5}\left(\theta -\theta_{0}\right)+c_{6}\;\mathrm{and}\;\chi_{\infty }=c_{7}\left(\theta -\theta_{0}\right)+c_{8}$$

where $c_{5}$, $c_{6}$, $c_{7}$, and $c_{8}$ are material constants.

### 2.5. Criterion for Viscoplasticity

The loading function to control the evolution of the viscoplastic deformation is given by:

(12) $$F=\sqrt{3J_{2}\left(\tau \right)}-A_{s}-A_{h}-Y,$$

with:

(13) $$A_{s}=\kappa_{s}\zeta_{s},\;A_{h}=\kappa_{h}\zeta_{h}\;\mathrm{and}\;Y=\kappa_{0}\left[1+c_{r}{\dot{h}}_{eq}^{m}+c_{\theta }\left(\theta -\theta_{0}\right)\right]$$

where $A_{s}$ and $A_{h}$ denote the thermodynamic driving forces associated, respectively, with the internal state variables $\zeta_{s}$ and $\zeta_{h}$; $\kappa_{s}$ and $\kappa_{h}$ are the respective internal stress moduli; *Y* denotes the temperature- and strain rate-dependent initial yield stress; ${\dot{h}}_{eq}=\sqrt{\frac{2}{3}}∥D∥$ denotes the equivalent strain rate; $\kappa_{0}$, $c_{r}$, *m*, and $c_{\theta }$ are the model parameters. The loading function *F* and the viscoplastic multiplier $\dot{\gamma }$ are subjected to the following Kuhn–Tucker consistent conditions:

(14) $$\dot{\gamma }\geq 0,\;F\leq 0,\;\mathrm{and}\;\dot{\gamma }F=0.$$

### 2.6. Temperature Evolution

As we mentioned above, the mechanical properties of amorphous polymers, such as the elastic modulus, yield stress, and flow stress, are significantly affected by the temperature. On the other hand, evolutions of the irreversible internal state variables, such as $h^{p}$, $\zeta_{s}$, and $\zeta_{h}$, give rise to an intrinsic dissipation and therefore a large amount of heat production during the viscoplastic deformation. Following the thermodynamic framework established for the inelastic material with internal state variables [34], the temperature evolution is formulated as:

(15) $$c\dot{\theta }=\rho \dot{r}-\nabla \cdot q+\omega \left(\sqrt{\frac{3}{2}}∥s∥\dot{\gamma }-A_{s}{\dot{\zeta }}_{s}-A_{h}{\dot{\zeta }}_{h}\right)$$

where *c* is the specific heat capacity, ρ is the mass density, *r* denotes the heat source, q denotes the heat flux, and ω is a Taylor–Quinney coefficient.

## 3. Calibration of the Model Parameters

In this section, we present the calibration procedure to identify the model parameters. First, we simplify the 3D constitutive equations into their 1D version. Then, we implement the simplified model into MATLAB by using an explicit integration scheme and calibrate the values of the model parameters by fitting experimental data. Finally, we compare the model predictions against the experimental data to validate the numerical efficiency and accuracy of the proposed model.

### 3.1. 1D Version of the Constitutive Equations

For the case of uniaxial loading, the Kirchhoff stress tensor τ and the deformation gradient F are given as:

(16) $$\tau =\tau \;e_{1}⊗e_{1}\;\mathrm{and}\;F=\lambda \;e_{1}⊗e_{1}+\lambda_{l}\;e_{2}⊗e_{2}+\lambda_{l}\;e_{3}⊗e_{3},$$

where τ and λ denote the uniaxial stress and stretch, $\lambda_{l}$ denotes the lateral contractions, and $e_{1},e_{2},e_{3}$ are the three orthonormal bases. Here, we excluded the spherical stress-strain response in Equation (6) and therefore assumed the deformation gradient isochoric, which gives the relation $\lambda_{l}=\sqrt{1/\lambda }$. Thus, the elastic and plastic deformation gradients are expressed as:

(17) $$\begin{array}{c} F^{e}=\lambda^{e}\;e_{1}⊗e_{1}+\sqrt{1/\lambda^{e}}\;e_{2}⊗e_{2}+\sqrt{1/\lambda^{e}}\;e_{3}⊗e_{3}, \end{array}$$

(18) $$\begin{array}{c} F^{p}=\lambda^{p}\;e_{1}⊗e_{1}+\sqrt{1/\lambda^{p}}\;e_{2}⊗e_{2}+\sqrt{1/\lambda^{p}}\;e_{3}⊗e_{3}, \end{array}$$

and the deviatoric stress tensor s is expressed as:

(19) $$s=\frac{2}{3}\tau \;e_{1}⊗e_{1}-\frac{1}{3}\tau \;e_{2}⊗e_{2}-\frac{1}{3}\tau \;e_{3}⊗e_{3}.$$

Under the uniaxial loading condition, the elastic Eulerian Hencky strain ${\bar{h}}^{e}$ is given as:

(20) $${\bar{h}}^{e}=\ln F^{e}=\ln\lambda^{e}\cdot T\;\mathrm{with}\;T=1\;e_{1}⊗e_{1}-\frac{1}{2}\;e_{2}⊗e_{2}-\frac{1}{2}\;e_{3}⊗e_{3}.$$

Thus, we obtain the following 1D stress-strain relation:

(21) $$s=2\mu \ln\lambda^{e}\cdot T\;\mathrm{and}\;\tau =3\mu \ln\lambda^{e}.$$

Then, by substitution of Equation (21) into Equation (7), we get the following viscoplastic flow rule under the uniaxial loading case:

(22) $${\tilde{D}}^{p}=\dot{\gamma }\cdot T.$$

### 3.2. Model Parameter Calibration

In order to calibrate the model parameters, we implemented the aforementioned constitutive equations into MATLAB by using an explicit integration scheme. The model parameters that need to be calibrated were classified into five categories: elasticity, yielding, softening, hardening, and heat, as listed in Table 1.

**Figure 1:**
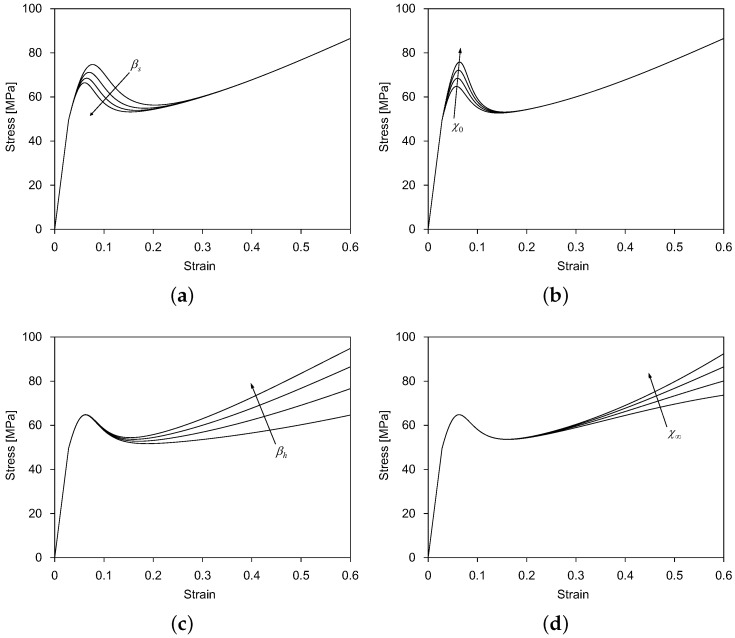
Effects of the model parameters (**a**) $\beta_{s}$, (**b**) $\chi_{0}$, (**c**) $\beta_{h}$, (**d**) $\chi_{\infty }$ on the softening and hardening behavior; arrows indicate the value increase of the respective parameters.

These model parameters were calibrated from the experimental data as follows:

- The reference elastic modulus $E_{0}$ was determined from the experimental stress-strain response at the reference temperature $\theta_{0}$. The model parameter $\Delta E_{\theta }$ was determined from the experimental stress-strain response at different temperatures. Poisson’s ratio ν and the thermal expansion coefficient α were obtained from the literature [26].
- The model parameters $\kappa_{0}$, $c_{r}$, *m*, and $c_{\theta }$ control the dependence of the initial yield stress on the strain rate and temperature. The model parameter $\kappa_{0}$ represents the initial yield stress at the minimum strain rate $\varepsilon_{min}$ and the reference temperature $\theta_{0}$, and it was determined approximately from the experimental stress-strain response at the strain rate of 0.0005 s^−1^ and the temperature of 25 °C. The model parameters $c_{r}$ and *m* were determined by fitting the exponential curve relationship between the yield stress and the logarithmic strain rate over the strain rate from 0.0005 s^−1^–8400 s^−1^. The model parameter $c_{\theta }$ was determined by fitting the linear relationship between the yield stress and the temperature over the temperature from −60 °C–120 °C. See the literature [25] for more details.
- The strain softening-associated model parameters $\kappa_{s}$, $c_{1}$, $c_{2}$, $c_{3}$, and $c_{4}$ control the evolution of the internal variable $\zeta_{s}$. According to Equation (9), $c_{1}$ and $c_{2}$ are used to compute the state variable $\beta_{s}$, while $c_{3}$ and $c_{4}$ are used to compute the state variable $\chi_{0}$. Figure 1a,b shows how the parameters $\beta_{s}$ and $\chi_{0}$ affect the strain softening behavior of amorphous polymers. $\kappa_{s}$, $c_{2}$, and $c_{4}$ were determined from the experimental stress-strain response at the reference temperature $\theta_{0}$, while $c_{1}$ and $c_{3}$ were determined from the experimental stress-strain response at different temperatures.
- The strain hardening-associated model parameters $\kappa_{h}$, $c_{5}$, $c_{6}$, $c_{7}$, and $c_{8}$ control the evolution of the internal variable $\zeta_{h}$. According to Equation (11), $c_{5}$ and $c_{6}$ are used to compute the state variable $\beta_{h}$, while $c_{7}$ and $c_{8}$ are used to compute the state variable $\chi_{\infty }$. Figure 1c,d shows how the parameters $\beta_{h}$ and $\chi_{\infty }$ affect the strain hardening behavior of amorphous polymers. $\kappa_{h}$, $c_{6}$, and $c_{8}$ are determined from the experimental stress-strain response at the reference temperature $\theta_{0}$, while $c_{5}$ and $c_{7}$ are determined from the experimental stress-strain response at different temperatures.
- The specific heat capacity *c*, the thermal conductivity *k*, and the mass density ρ were obtained from the literature [26]. The Taylor–Quinney factor ω was determined from the temperature change experimentally measured by the thermal imager. The reference temperature $\theta_{0}$ took the room temperature of 25 °C.

Finally, the model parameters were calibrated from the experimental data presented in the literature [25] and are listed in Table 2.

### 3.3. Model Validation

After calibrating the model parameters, we implemented the proposed constitutive model into the Finite Element software ABAQUS/Explicit by means of the user-defined material subroutine VUMAT. The numerical efficiency and robustness of the model were validated against the experimental data presented in the literature [25,35].

**Figure 2:**
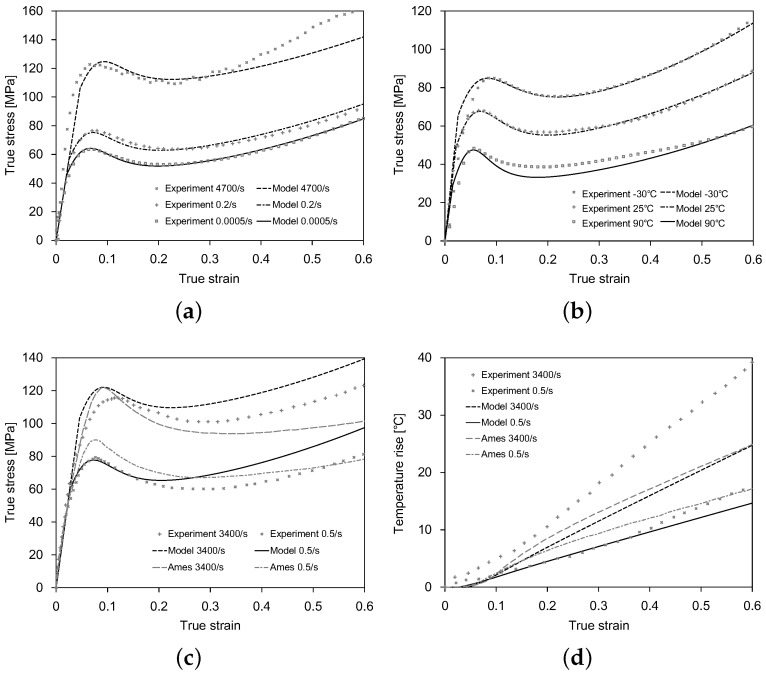
Model validations against the experimental data: (**a**) stress-strain curves at different strain rates; (**b**) stress-strain curves at different temperatures; (**c**) stress-strain curves and (**d**) temperature rises compared to the experimental data of [35] and simulation results of [4].

We simulated a single hexahedral element, with dimensions of 1 mm × 1 mm × 1 mm, under uniaxial loading at different strain rates and temperatures. The C3D8RT element was adopted to capture the thermomechanically-coupled behavior of polycarbonate. Figure 2a shows the comparisons of the model predictions to the experimental data [25] at a temperature of 25 °C and strain rates of 0.0005 s^−1^, 0.2 s^−1^, and 4700 s^−1^, while Figure 2b at a strain rate of 0.0005 s^−1^ and temperatures of −30 °C, 25 °C, and 90 °C. Figure 2c shows the comparisons of the model predictions to the experimental data [35] and the numerical predictions of [4] at strain rates of 0.5 s^−1^ and 3400 s^−1^, while Figure 2d gives the corresponding temperature rises. Overall, the model predictions agreed reasonably well with the experimental data by capturing the prime characteristics of amorphous glassy polymers, such as the strain rate and temperature-dependent yield transition, the strain softening and hardening, the large viscoplastic flow deformation, and the heat production.

## 4. Three-Point Bending

In this section, to illustrate the superiority of the proposed model when dealing with finite deformation boundary value problems, an example of a polycarbonate beam subjected to three-point bending is considered. First, we conducted three-point bending tests on polycarbonate beams. Then, we carried out FE simulations of the three-point bending tests using the proposed constitutive model and compared the model predictions to the experimental data.

**Figure 3:**
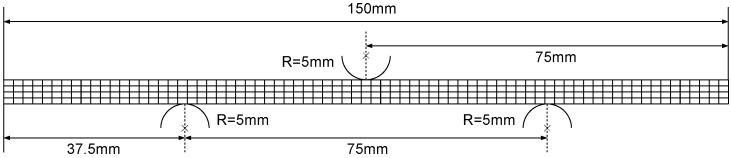
Geometry, dimension and mesh of the three-point bending model.

The geometry, the dimension and mesh of the three-point bending model are shown in Figure 3, of a width of 20 mm and six different thicknesses of 1.0 mm, 1.5 mm, 2.0 mm, 3.0 mm, 3.75 mm, and 4.92 mm. The three-point bending tests were conducted on the CRIMS DNS100 universal testing machine from CRIMS Ltd., Jilin, China. During the tests, the polycarbonate beam rested on two roller supports, and the top roller loaded downward, with a loading rate of 5 mm/min and a maximum displacement of 20 mm. The FE simulations of the tests were carried out in ABAQUS/Explicit, and the polycarbonate beam was meshed using coupled temperature-displacement reduced integration hexahedral elements (C3D8RT), while the rollers were analytically rigid. During the simulation, the two roller supports were fully constrained; a rigid body motion was applied on the top roller; an initial temperature field was prescribed on the beam; and the surface-to-surface contact was defined between the rollers and beam.

**Figure 4:**
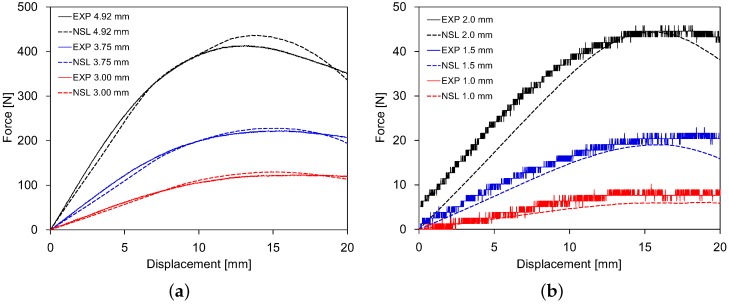
Force-displacement curves obtained in the three-point bending of polycarbonate beam for six different thicknesses: experimental data (EXP) and numerical simulations (NSL).

Figure 4 shows the force-displacement response of the polycarbonate beam subjected to three-point bending. Comparisons between the numerical results and the experimental data are presented for six different beam thicknesses of 1.0 mm, 1.5 mm, 2.0 mm, 3.0 mm, 3.75 mm, and 4.92 mm. The solid curves represent the experimental data, while the dashed curves represent the numerical results. It is noted that the fluctuations on the curves, especially for the three thinner thicknesses in Figure 4b, were due to the precision decline of the testing system when contact force was relatively small. Overall, the numerical results agreed well with the experimental data by capturing structural responses, such as initial linearity, nonlinear yield transition, and post-yield softening. Through these comparisons, we demonstrated that our proposed model can reasonably represent the thermomechanical behavior of an amorphous glassy polymer undergoing large strains and rotations.

**Figure 5:**
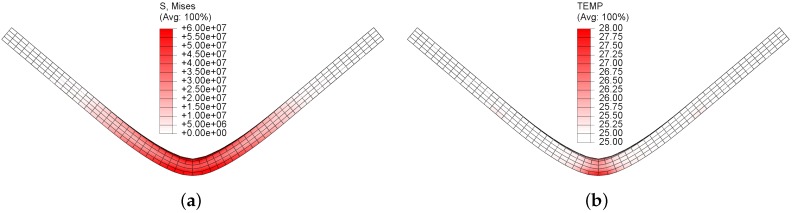
von Mises stress (**a**) and temperature (**b**) contour plots when the polycarbonate beam undergoes large deformation and rotation.

Figure 5 shows the von Mises stress and temperature contour plots on the polycarbonate beam at the maximum deformation. As can be seen from the figure, the strain and rotation on the beam was relatively large. The von Mises stress reached approximate 60 MPa at the central part of the beam, and the elements at the top layer were subjected to compressive loadings, while the bottom layer was subjected to tensile loadings. At such a stress level, the material had entered the viscoplastic regime. According to Equation (15), the intrinsic dissipation due to the viscoplastic deformation will partially convert to heat and therefore lead to a temperature rise on the beam, as shown in Figure 5b. Here, the maximum temperature rise was around 3 °C.

## 5. Gas-Blow Forming

**Figure 6:**
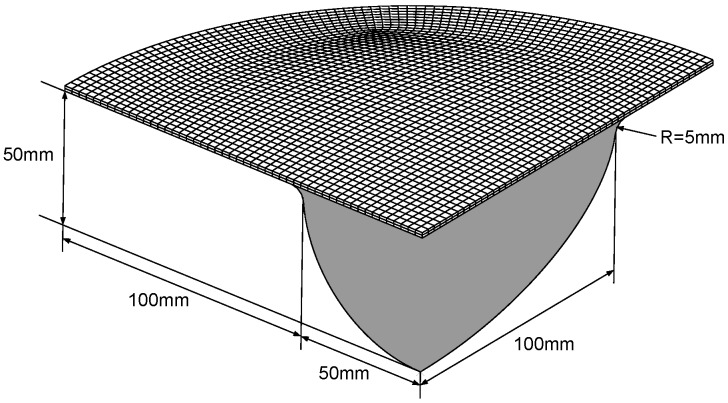
Finite element model of a quarter of the forming die and polycarbonate sheet.

In order to show the model’s capability of capturing the intricate behavior of amorphous glassy polymer during the warm mechanical process, we applied the model to simulate the gas-blow forming process of the polycarbonate sheet, which underwent large viscoplastic deformation at a temperature slightly lower than the glassy transition temperature. Figure 6 shows the analytical model adopted in the simulation. It is noted that, in consideration of the structural symmetry, we only created a quarter of the entire model. The element type for the sheet was C3D8RT (temperature-displacement coupled element), and the forming die was R3D4 (discrete rigid element). The boundary conditions and loading are given as follows: the side edges of the sheet were fully restricted; a uniform temperature was initially prescribed on the sheet; and the upper surface of the sheet was loaded with an increasing gas pressure.

**Figure 7:**
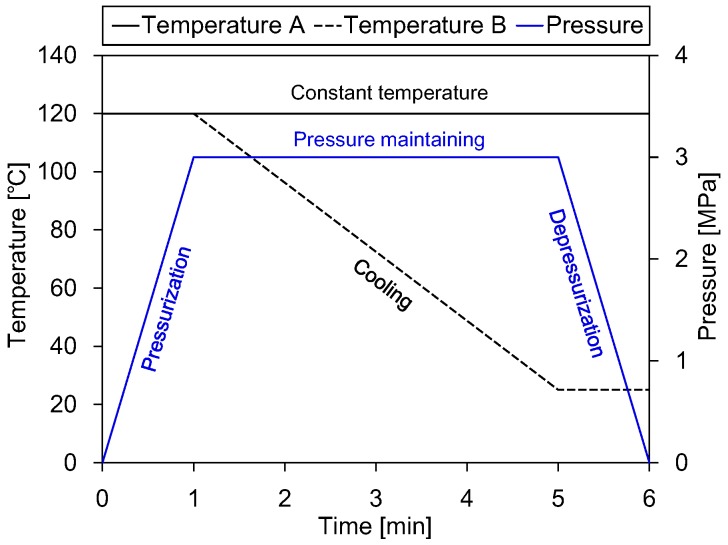
Thermomechanical loading cases considered in the gas-blow forming simulation.

The thermomechanical loading cases considered in the gas-blow forming simulations are shown in Figure 7. Gas pressure was increased from zero to 3 MPa in the first minute (pressurization stage), then kept constant in the next four minutes (pressure-maintaining stage) and finally decreased back to zero in the last minute (depressurization stage). For the temperature, a constant value of 120 °C was kept throughout the simulation in the first loading case, while decreasing from 120 °C–25 °C at the pressure maintaining stage in the second loading case.

Table 3 shows the deformations of the polycarbonate sheet at gas pressure levels of 1.0 MPa, 2.0 MPa, and 3.0 MPa during the pressurization process and the final configuration after depressurization, which adequately illustrates the gas-blow forming process of the polycarbonate sheet. The solid black curves represent the forming die, while the gray regions represent the polycarbonate sheet. Both major-axis and minor-axis views are presented in the table. During the pressurization process, the central part of the sheet deformed downward and approached the bottom of the forming die with the increasing gas pressure. When the gas pressure reached the maximum value of 3.0 MPa, the polycarbonate sheet filled up the cavity of the forming die, except small gaps at both sides of the major axis, indicating that these areas were the most difficult parts for the sheet to contact the forming die. Finally, when the gas pressure was completely released, the polycarbonate sheet displayed a slight spring back due to the elastic unloading.

**Figure 8:**
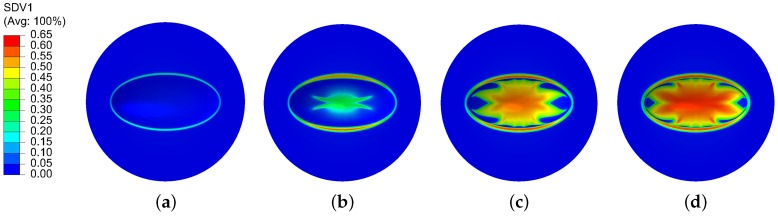
Viscoplastic strain contour plot on the deformed polycarbonate sheet at gas pressure levels of (**a**) 1.0 MPa, (**b**) 1.5 MPa, (**c**) 2.0 MPa, and (**d**) 3.0 MPa.

Figure 8 shows the viscoplastic strain contour plot on the deformed polycarbonate sheet at gas pressure levels of 1.0 MPa, 1.5 MPa, 2.0 MPa, and 3.0 MPa. As we can see from the figure, the viscoplastic strain firstly took place along the edge of the ellipse when the gas pressure was increased to 1.0 MPa. Then, with the increasing gas pressure, the central part of the sheet began to display viscoplasticity, and the deformed area gradually expanded outward. Finally, when the gas pressure reached the maximum value of 3.0 MPa, the viscoplastic strain contour plot formed a butterfly shape. The maximum viscoplastic strain was around 65%, which had definitely entered the finite-strain regime.

**Figure 9:**
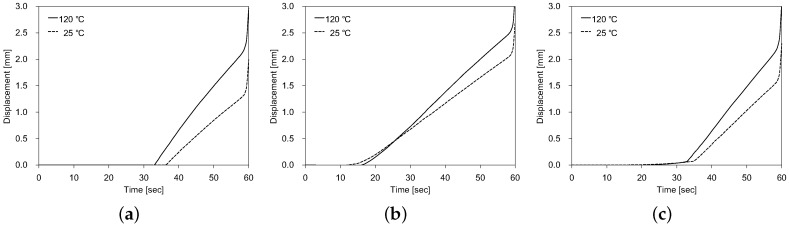
Spring back displacement of the polycarbonate sheet during the depressurization process in both loading cases: (**a**) center point, (**b**) midpoint of major semiaxis, and (**c**) midpoint of minor semiaxis.

Figure 9 shows the spring back displacement of the polycarbonate sheet during the depressurization process in both loading cases. Here, three representative points were considered: center point, midpoint of major semiaxis, and midpoint of minor semiaxis. As we mentioned above, in the first loading case, the temperature was maintained at 120 °C during the depressurization process; while in the second loading case, the temperature was cooled down from 120 °C–25 °C before the depressurization process. According to Equation (4), the elastic modulus of amorphous glassy polymer strongly depends on the temperature. Specifically, when the temperature is decreased, it grows proportionally. As depressurization is approximately an elastic unloading process, the larger elastic modulus in the second loading case produced a smaller spring back deformation, as shown in Figure 9. For a 2 mm-thick polycarbonate sheet, the maximum spring back deformation was about 3 mm.

## 6. Conclusions

In this paper, we developed a finite-strain and thermomechanically-coupled constitutive model to represent the large viscoplastic deformation of amorphous glassy polymers. The model began with a kinematic hypothesis that the deformation gradient was multiplicatively decomposed into elastic, viscoplastic, and thermal components. The Eulerian Hencky strain rate and Jaumann rate of Kirchhoff stress were adopted to formulate the model with respect to the spatial configuration. Constitutive equations involving hyperelasticity, the viscoplastic flow rule, strain softening and hardening, the criterion for viscoplasticity, and temperature evolution were derived with the finite-strain framework. In order to validate the numerical efficiency and stability of the proposed model, we first implemented the model in numerical software to calibrate the model parameters from the experimental data obtained in uniaxial tensile tests. Then, we carried out three-point bending tests on a polycarbonate beam and the corresponding simulations and compared the predicted results to the experimental data. Finally, we simulated a gas-blow forming process of a polycarbonate sheet to demonstrate the capability of the proposed model, which can adequately represent the intricate thermomechanical behavior of amorphous glassy polymer under large deformation.

References

## Figures and Tables

**Table 1:** Model parameters that need to be calibrated.

Elasticity: $E_{0}$, $\Delta E_{\theta }$, ν, α;
Yielding: $\kappa_{0}$, $c_{r}$, *m*, $c_{\theta }$;
Softening: $\kappa_{s}$, $c_{1}$, $c_{2}$, $c_{3}$, $c_{4}$;
Hardening: $\kappa_{h}$, $c_{5}$, $c_{6}$, $c_{7}$, $c_{8}$;
Heat: *c*, *k*, ρ, ω, $\theta_{0}$.

**Table 2:** Model parameters used in the present simulations.

Categories	Parameters	Constants	Values
Elasticity	*E* (MPa)	$E_{0}$ (MPa)	2271
		$\Delta E_{\theta }$ (MPa/K)	−5.4
	ν		0.4
	α (K^−1^)		$6.8\times 10^{-5}$
Yielding		$\kappa_{0}$ (MPa)	35.16
		$c_{r}$ (s)	0.746
		*m*	0.118
		$c_{\theta }$ (K^−1^)	$-8.6\times 10^{-3}$
Softening ($\zeta_{s}$)	$\kappa_{s}$ (MPa)		130.45
	$\beta_{s}$	$c_{1}$	0.036
		$c_{2}$ (K^−1^)	10.24
	$\chi_{0}$	$c_{3}$	0.105
		$c_{4}$ (K^−1^)	26.86
Hardening ($\zeta_{h}$)	$\kappa_{h}$ (MPa)		82.36
	$\beta_{h}$	$c_{5}$	−0.012
		$c_{6}$ (K^−1^)	3.12
	$\xi_{\infty }$	$c_{7}$	1.45
		$c_{8}$ (K^−1^)	6.46
Heat	*c* (J/(kg · K))		$1.63\times 10^{3}$
	*k* (W/(m · K))		0.19
	ρ (kg/m^3^)		$1.19\times 10^{3}$
		ω	0.86
		$\theta_{0}$ (K)	298

**Table 3:** Deformations of the polycarbonate sheet at gas pressure levels of 1.0 MPa, 2.0 MPa, and 3.0 MPa and the spring back configuration after depressurization.

Gas Pressure	Major Axis	Minor Axis
Pressurization 1.0 MPa	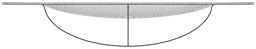	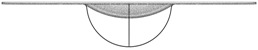
Pressurization 2.0 MPa	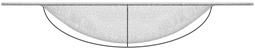	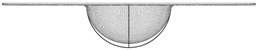
Pressurization 3.0 MPa	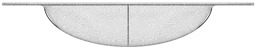	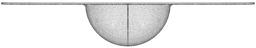
Depressurization 0.0 MPa